# Unveiling *Facklamia*: detection of an emerging microbe in the skin microbiome of patients with filarial lymphedema

**DOI:** 10.3389/fcimb.2025.1624288

**Published:** 2025-07-31

**Authors:** Danapriyaa Dharmalingam, Janani Semalaiyappan, Sankari Thirumal, Vijesh Sreedhar Kuttiatt

**Affiliations:** Unit of Clinical and Molecular Medicine, ICMR-Vector Control Research Centre, Puducherry, India

**Keywords:** *Facklamia*, filarial lymphedema, skin microbiome, opportunistic pathogen, 16S rRNA-based metagenomics

## Abstract

*Facklamia* is an emerging pathogen in human beings and only a few clinical cases were reported in the literature. We detected the presence of this unusual microbe among the skin flora of three patients with filarial lymphedema in a 16S rRNA-based metagenomic study on the skin microbiome. To our knowledge, this is the first report of detection of this microbe in patients affected with filarial lymphedema. Further investigations are required to elucidate the role of *Facklamia* in secondary skin and soft tissue infection of filarial lymphedema patients.

## Introduction

Lymphatic filariasis is a neglected tropical parasitic disease (skin NTD) caused by the filarial parasites and transmitted by *Culex* species of mosquitos. Filarial infection results in limb lymphedema leading to elephantiasis, a worldwide health concern, especially in tropical and subtropical countries. Patients with lymphedema are prone to recurrent skin and soft tissue infection and experience frequent adenolymphangitis (ADL) episodes. The lymph stasis and the associated skin changes alter the skin microflora, and this may play a major role in frequent ADL attacks (Shenoy, 2008; Rajasekaram et al., 2017). A study from rural Ghana has previously explored the skin flora in individuals with filarial lymphedema, providing early insights into microbial community shifts associated with disease (Kwarteng et al., 2022). In a recent study using 16S rRNA-based metagenomics, we noted differences in the skin microbiome of patients affected with filarial lymphedema compared to healthy volunteers (Manavalan et al., 2025). We employed a 16S rRNA-based metagenomic technique, which has the advantage of detecting difficult-to-culture microbes for the detection of skin microflora of patients affected with filarial lymphedema. Some unusual microbes were detected in the skin of patients affected with filarial lymphedema, including members of the *Aerococcus, Eremococcus, Enhydrobacter, Macrococcus, Terribacillus, Dermabacter, Arcanobacterium, Mannheimia* genera and *Facklamia* were one among them (Manavalan et al., 2025). In this report, we specifically describe the detection of *Facklamia* spp., in three patients with filarial lymphedema enrolled in the study by Manavalan et al.

## Materials and methods

This study was conducted at the Filariasis Management Clinic of ICMR(Indian Council of Medical Research)-Vector Control Research Centre (VCRC), and the methodology has been described in detail previously (Manavalan et al., 2025). Briefly, the study participants were patients with grade III & IV filarial lymphedema (n=10) and healthy individuals (n=10) who were staff of VCRC. Participants (both patients and controls) with other skin diseases or systemic illnesses known to cause immunodeficiency, such as HIV infection, diabetes mellitus, chronic kidney disease, cancer, or those receiving immunosuppressive therapy, were excluded from the study based on their past medical history, which was collected during recruitment. Ethical approval was obtained from the Institutional Human Ethics Committee of VCRC (IHEC-0222/N/F), and written informed consent was obtained from all participants and patients for participation in the study. Two skin swabs were collected from the legs of each participant. One swab was used for overnight culture to isolate viable skin microbiota. The second swab was preserved under sterile conditions as a backup and was intended for use only if the primary sample failed quality control during downstream sequencing analysis. The V3-V4 region of the 16S rRNA gene was amplified from the DNA extracted from the cultures and subjected to sequencing on the Illumina NovaSeq platform. Raw sequencing reads were initially processed to eliminate low-quality sequences. High quality reads were analysed using QIIME2 (version 2022.2). Sequence alignment was carried out against the SILVA and Green Genes RNA reference databases. Chimeras were filtered using USEARCH and UCLUST algorithms, and Operational Taxonomic Units (OTUs) were clustered at a 97% sequence similarity threshold for taxonomic assignment (Manavalan et al., 2025). The sequences obtained were submitted to GenBank (SAMN44092209, SAMN44092210, SAMN44092214) under the Bioproject ID PRJNA1170177.

**Figure 1:**
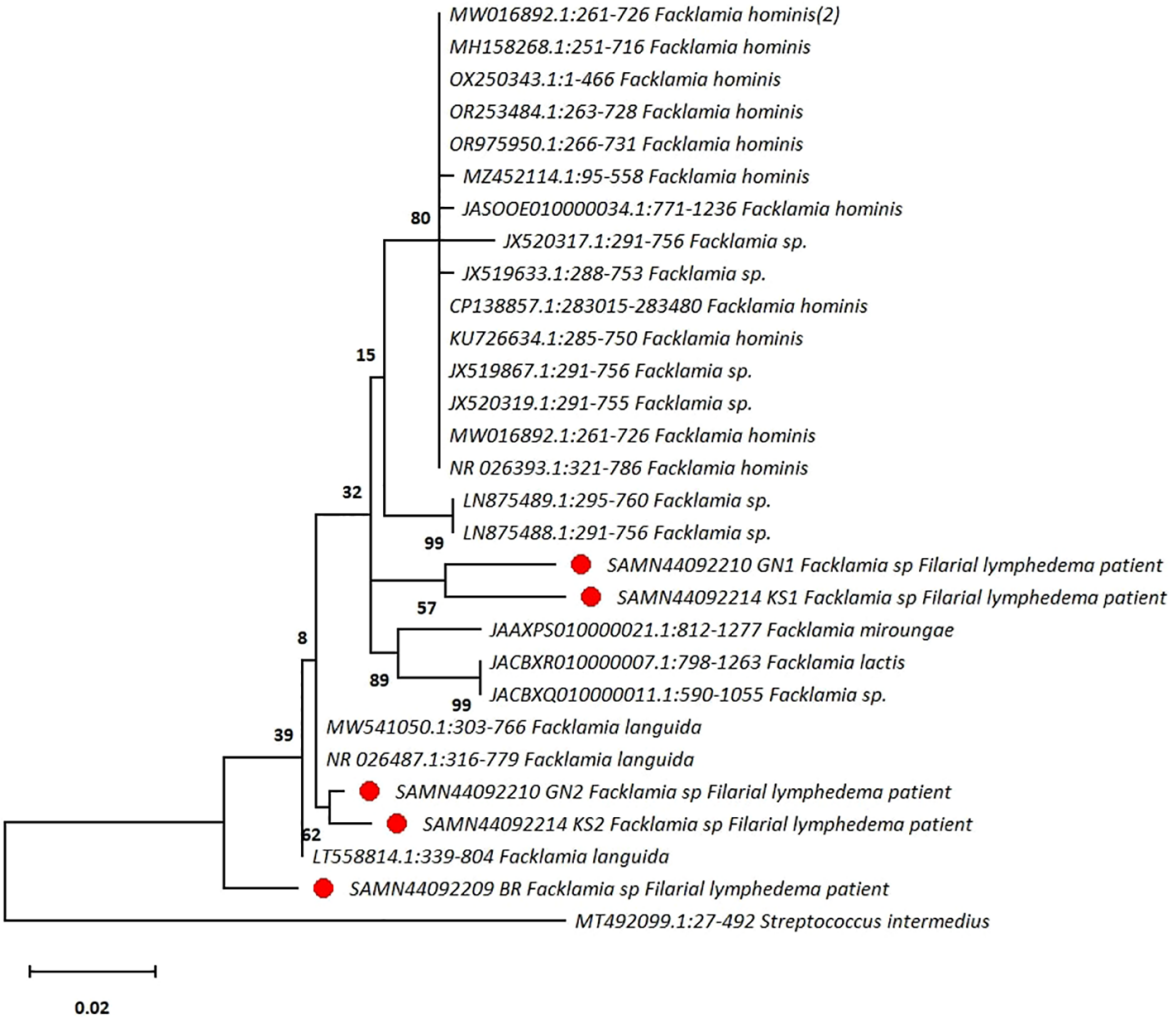
The phylogenetic tree shows that sequences obtained from patients with filarial lymphedema are clustered with *Facklamia* spp. A sequence from *Streptococcus intermedius*, a member of the viridans group streptococci, is used as an outgroup. The tree is typically drawn to scale and indicates the number of substitutions per site. (●Highlighted in red circle represent the presence of *Facklamia* species among the skin flora of the affected legs of three patients with filarial lymphedema in our study).

Nucleotide BLAST search was performed using standard metrics to identify matches from the from NCBI GenBank database. Sequences matching the query sequences were retrieved from NCBI GenBank database and a phylogenetic tree was constructed in MEGA Ver 11.0 using the maximum likelihood method based on the Tamura-Nei model with 500 bootstrapping iterations to confirm that the sequences belong to *Facklamia* spp (Tamura et al., 2021). For this purpose, the 16SrRNA sequence of a *Streptococcus intermedius*, a member of the viridans group streptococci, was used as an outgroup (**Figure 1**).

## Results

Using EZbiocloud 16S rRNA database, five sequences from the skin flora of three patients with filarial lymphedema (**Table 1**) were found to belong to the genus *Facklamia* (**Table 1**). However, *Facklamia* bacterial sequences were not detected in any of the ten healthy individuals. In the phylogenetic tree, two sequences clustered with *Facklamia languida* species and showed >99.5% sequence identity with *Facklamia* sequences in the GenBank. Among the remaining three sequences, two formed a distinct cluster, and the third sequence was identified as an ancestor to all the available *Facklamia* sequences. These sequences were from three patients with Biosample ID SAMN44092209, SAMN44092210GN1 and SAMN44092214KS1, respectively. The percentage of sequence identity of three sequences was <97% with other *Facklamia* sequences in the GenBank database, which indicate these may belong to a new species of *Facklamia*, as the sequence identity of 97% threshold has been the widely accepted standard for delineation at the species level using 16S rRNA-based next generation sequencing methods (Kim et al., 2014).

**Table 1:** Patient characteristics.

S.no	Patient ID	Age (Yrs)	Sex	Lymphedema grade	Associated conditions	Contact with animals
1	KS	55	Male	IV	Nil	Nil
2	BR	58	Male	III	Hypertension	Nil
3	GN	50	Male	IV	Nil	Nil

## Discussion

In the current study, the presence of *Facklamia* was noted in three patients with filarial lymphedema and in none of the healthy controls. All skin swabs were collected from intact skin, and we avoided ulcerated or visibly open lesions, to focus on resident skin flora rather than wound-associated microbiota. Participants did not exhibit adenolymphangitis (ADL) during sampling, reducing confounding related to acute infections. *Facklamia* was detected after overnight culturing of skin swabs, with DNA extracted from the cultured microbial biomass, indicating the presence of viable, culturable organisms rather than environmental DNA.

*Facklamia* was reported to be a resident bacterium in the female urethra and vagina (Rahmati et al., 2017). They are gram-positive cocci that can be found in both groups and pairs. They are catalase-negative, alpha-hemolytic, and facultatively anaerobic. *Facklamia* was reported to resemble viridans streptococci on 5% of sheep blood agar (Collins et al., 1997; Hoyles, 2014). Biochemical tests and analysis of the 16S rRNA gene sequencing or MALDI-TOF are required for precise characterization as it is challenging to differentiate them from other cocci (Pérez-Cavazos et al., 2022). Technologies like 16S rRNA-based metagenomic sequencing has the added advantage of detecting unculturable pathogens.

The sequences obtained from the patient samples were identified as belonging to the genus *Facklamia* and not to the viridans group of streptococci, as sequences from the viridans group shared only 86–87% identity with the *Facklamia* sequences in our study. There were a few earlier studies which distinguish *Facklamia* from viridans streptococci, as it was often misidentified as *Streptococcus* (LaClaire and Facklam, 2000). It is important to identify *Facklamia* to the species level and to distinguish it from the viridans group of streptococci due to its varied antimicrobial susceptibility profile (LaClaire and Facklam, 2000). *F. languida* was reported to possess elevated MICs (Minimum Inhibitory Concentration) to carbapenem antibiotics compared to the viridans group of streptococci. Similarly, *F. ignava* was found to possess elevated MICs to penicillin, and both *F. ignava* and *F. languida* were reported to have elevated MICs to erythromycin, cefotaxime, trimethoprim-sulfamethoxazole, and clindamycin.

There are currently six known species in the *Facklamia* genus, four of which, *F. hominis, F. ignava, F. sourekii, and F. languida*, were reported to be linked to human infection. They were isolated from different clinical specimens which include skin, bone, gallbladder, chorioamnionitis swabs, blood, joint, abscess, hidradenitis suppurativa, vaginal, urinary tract and cerebrospinal fluid (Healy et al., 2005; Corona et al., 2014; Rahmati et al., 2017; Mostafa et al., 2019; Basal et al., 2021). *Facklamia hominis* was the most commonly reported species identified in clinical specimens (Rahmati et al., 2017; Pérez-Cavazos et al., 2022). However, in our study, *Facklamia languida* species was found among the skin flora of affected legs of three patients affected with filarial lymphedema. We presume that unusual microbes like *Facklamia* colonize the skin due to lymph stasis and localized immunodeficiency associated with chronic lymphedema. Further detailed studies are required to better characterize these emerging microbes, their pathogenicity, and their antimicrobial sensitivity susceptibility.

Though *Facklamia* spp. have been associated with invasive infections and have been isolated in culture from clinical specimens, the limited number of documented cases, the simultaneous presence with other bacteria, and the common occurrence of substantial co-morbidities in patients make it difficult to infer their true pathogenicity.

It can be considered a facultative pathogen or an opportunistic pathogen as it is present in healthy individuals with only occasional reports of invasive infection. Therefore, further research is necessary to delineate the role of *Facklamia* and its association in the pathogenesis of filarial lymphedema.

Limitations: In this study, the methodology adopted was sequencing the V3-V4 region of the 16S rRNA gene. However, accurate species-level identification is challenging with Illumina-based short-read sequencing targeting the V3–V4 or V4 regions due to the high similarity of 16S regions among bacterial species. Short-read 16S sequencing has limited resolution for species-level identification, necessitating the use of long-read sequencing technologies (PacBio or Oxford Nanopore) for more definitive results and may be carried out in future studies. Small sample size and low OTU counts are other limitations. However, detection of the presence of this microbe in the skin of patients with filarial lymphedema and not in healthy controls in this study, warrants further investigation in larger, well-powered cohorts to confirm its significance and generalizability.

## Data Availability

The datasets presented in this study can be found in online repositories. The names of the repository/repositories and accession number(s) can be found below: https://www.ncbi.nlm.nih.gov/genbank/, PRJNA1170177.
